# The Effects of a Very-Low-Calorie Ketogenic Diet on the Intestinal Barrier Integrity and Function in Patients with Obesity: A Pilot Study

**DOI:** 10.3390/nu15112561

**Published:** 2023-05-30

**Authors:** Michele Linsalata, Francesco Russo, Giuseppe Riezzo, Benedetta D’Attoma, Laura Prospero, Antonella Orlando, Antonia Ignazzi, Martina Di Chito, Annamaria Sila, Sara De Nucci, Roberta Rinaldi, Gianluigi Giannelli, Giovanni De Pergola

**Affiliations:** 1 Functional Gastrointestinal Disorders Research Group, National Institute of Gastroenterology IRCCS “S. de Bellis”, 70013 Castellana Grotte, Italy; michele.linsalata@irccsdebellis.it (M.L.); giuseppe.riezzo@irccsdebellis.it (G.R.); benedetta.dattoma@irccsdebellis.it (B.D.); laura.prospero@irccsdebellis.it (L.P.); antonella.orlando@irccsdebellis.it (A.O.); antonia.ignazzi@irccsdebellis.it (A.I.); 2 Center of Nutrition for the Research and the Care of Obesity and Metabolic Diseases, National Institute of Gastroenterology IRCCS “Saverio de Bellis”, 70013 Castellana Grotte, Italy; dichitomartina@gmail.com (M.D.C.); annamaria.sila@irccsdebellis.it (A.S.); sara.denucci@irccsdebellis.it (S.D.N.); roberta.rinaldi@irccsdebellis.it (R.R.); giovanni.depergola@irccsdebellis.it (G.D.P.); 3 Scientific Direction, National Institute of Gastroenterology IRCCS “S. de Bellis”, 70013 Castellana Grotte, Italy; gianluigi.giannelli@irccsdebellis.it

**Keywords:** obesity, intestinal barrier, intestinal permeability, ketogenic diet, dysbiosis, inflammation

## Abstract

The very-low-calorie ketogenic diet (VLCKD) is effective and safe for obese individuals, but limited information exists on its impact on the intestinal barrier. This study analyzed the effects of 8 weeks of VLCKD on 24 obese patients (11M/13F). Carbohydrate intake was fixed at 20–50 g/day, while protein and lipid intake varied from 1–1.4 g/kg of ideal body weight and 15–30 g per day, respectively. Daily calorie intake was below 800 kcal. The lactulose–mannitol absorption test assessed small intestinal permeability. Multiple markers, such as serum and fecal zonulin, fatty acid-binding protein, diamine oxidase concentrations, urinary dysbiosis markers (indican and skatole), and circulating lipopolysaccharide levels, were analyzed. Inflammation markers (serum interleukin 6, 8, 10, and tumor necrosis factor-α concentrations) were also evaluated. The results showed significant reductions in weight, BMI, and waist circumference post-diet. However, the lactulose–mannitol ratio increased by 76.5%, and a significant increase in dysbiosis markers at the end of the diet occurred. This trend was particularly evident in a subgroup of patients. Despite initial benefits, the VLCKD might negatively affect the intestinal barrier function in obese patients, potentially worsening their compromised intestinal balance.

## 1. Introduction

Obesity is a metabolic disorder characterized by long-term, low-grade inflammation, leading to significant impairments in health status with severe comorbidities [1]. In patients with obesity, several studies have pointed to modifications in gastrointestinal (GI) functions, including alterations in the integrity and function of the intestinal barrier. 

The intestinal barrier represents a functional unit that allows the absorption of nutrients and prevents the penetration of unwanted, often dangerous, macromolecules [2]. In addition, it has been suggested that intestinal permeability (IP) may be related to metabolic disorders, including obesity, gut microbial balance, low-grade inflammation, and diet.

According to animal studies, the connection between the gut’s adipose tissue and the gut’s brain is crucial for preserving energy balance and is hampered by metabolic abnormalities. There is growing evidence that the intestinal microflora causes the low-grade inflammation that results in intestinal barrier dysfunction, increases its permeability, and permits endotoxemia, which is one of the main factors in the emergence of metabolic inflammation and insulin resistance [3,4]. The alteration of the intestinal microbiota composition and the intestinal barrier function can directly or indirectly influence the production and secretion of intestinal endocrine hormones, thus triggering metabolic diseases.

In this respect, it is known that particular foods and excessive caloric intake can determine changes in the intestinal microbiota [5] and epithelial damage, leading to increased IP and translocation of the luminal content to the underlying mucosa [6]. Impairment of the intestinal barrier has been observed in patients with morbid obesity, which is also associated with an increased inflammatory response in the systemic compartment [7]. 

Concerning the diet as a treatment to reduce body weight, the ketogenic diet (KD), with its drastic carbohydrate decrease, is now common for weight loss and, in particular, a very-low-calorie ketogenic diet (VLCKD) is regarded as an efficient and secure therapeutic intervention for people affected by obesity [8,9].

Many studies have also shown the benefits of VLKD on body composition, metabolic profile, inflammation and oxidative stress gene expression in people with obesity [10,11]. 

Moreover, unaffected by changes in the symptoms of Non-alcoholic Fatty Liver Disease (NAFLD) such as obesity, fat mass, insulin resistance, lipids, and blood pressure, VLCKD is an effective treatment for this condition [12]. However, there are no data in the literature about the long-term effects of VLKD on intestinal barrier homeostasis, specifically in subjects with obesity. Previously, in an animal model of irritable bowel syndrome (IBS), we observed that KD affected glucose metabolism and intestinal membrane permeability, with an overexpression of the glucose transporter GLUT1 and tight junction proteins [13].

In humans, intestinal barrier function can be evaluated using non-invasive methods such as the urinary assay of non-absorbable sugars of various sizes (e.g., lactulose—Lac, mannitol—Man, and sucrose—Suc). Lac is a disaccharide that provides information on the paracellular pathway and tight junction (TJ) integrity, while Man is a monosaccharide that is thought to reflect the transcellular route. These sugars are degraded by bacteria, mostly those present in the colon. Therefore, their 0–5 h urinary fractions are currently used as markers for small IP (s-IP), and the Lac to Man ratio represents humans’ most-used functional measurement of s-IP, as it eliminates factors related to uptake, distribution, or excretion. The third sugar, Suc, is a disaccharide hydrolyzed by the enzyme sucrase in the jejunum, and it can represent an index of gastroduodenal permeability. This sugar absorption test (SAT) is now widely used for diagnosing “leaky gut” syndrome, defined as a gut mucosal barrier dysfunction that results in abnormally increased intestinal permeability [14].

The evaluations of fecal and serum levels of zonulin, a protein that reversibly modulates s-IP by altering the TJ interaction [15], are among the additional assays to assess the function of the GI barrier. Additionally, because they are immediately released in response to the cell membrane’s altered integrity and then manifest in the bloodstream, the intestinal fatty acid-binding protein (I-FABP) [16] and diamine oxidase (DAO) [17] are now regarded as potential markers for intestinal epithelial barrier health [18].

To our knowledge, no research has previously been performed to evaluate, in humans, the possible modifications occurring in the gut barrier homeostasis following a VLCKD. Based on these premises, the present study aimed to assess, in patients with obesity without impaired small intestinal permeability, the effects of 8 weeks of VLCKD on the integrity and function of the intestinal barrier. For these purposes, the circulating and urinary markers mentioned above were investigated. Additionally, the inflammatory status was assessed by measuring the circulating levels of interleukin 6, 8, and 10 (IL-6, IL-8, IL-10), and tumor necrosis factor-α (TNF-α). Finally, the urinary markers of dysbiosis indican and skatole and the lipopolysaccharide (LPS) circulating levels were also evaluated.

## 2. Materials and Methods

### 2.1. Study Design and Population

The Center of Nutrition for the Research and the Care of Obesity and the Metabolic Diseases together with the Functional Gastrointestinal Disorders Research Group afferent to the National Institute of Gastroenterology IRCCS “Saverio de Bellis”, Castellana Grotte (Ba) Italy, performed this pilot study. 

Patients with obesity, aged 18–65 yrs., with a body mass index (BMI) higher than 30 kg/m^2^, underwent a check of medical history, an anthropometric assessment, physical examination, and laboratory tests. To obtain a homogenous group, only patients with obesity responding to the exclusion criteria (see below) and without IBS and impaired small intestinal permeability, defined as Lac to Man ratio ≥ 0.030 [14], were enrolled.

During the medical history collection, specific questions were asked concerning the smoking habits and the daily alcohol consumption, according to the American and European recommendations, that is, two glasses of alcohol per day for male patients and one glass of alcohol per day for female patients [19,20], defining a threshold of 30 g/day for men and 20 g/day for women. 

The exclusion criteria included the contraindications to begin a VLCKD [8,9,10,11], such as hypersensitivity to components contained in meal replacement products, type 1 diabetes mellitus, history of cerebrovascular and cardiac diseases, respiratory insufficiency, severe GI diseases (i.e., inflammatory bowel disease, autoimmune diseases, cancer), chronic kidney disease characterized by an estimated glomerular filtration rate < 60, psychiatric issues, or pregnancy and lactation. Additional exclusion criteria were eating disorders and other serious mental illnesses, liver failure, substance abuse, frail elderly patients, active/severe infections, and rare disorders such as porphyria or deficiency of carnitine or carnitine–palmitoyl transferase or carnitine–acylcarnitine translocase or pyruvate carboxylase, and disorders of mitochondrial fatty acid oxidation. Finally, all patients enrolled in the study were instructed not to take any drugs, probiotics, vitamins, or other supplements. Supplements had to be discontinued 15 days before starting the diet.

**Figure 1:**
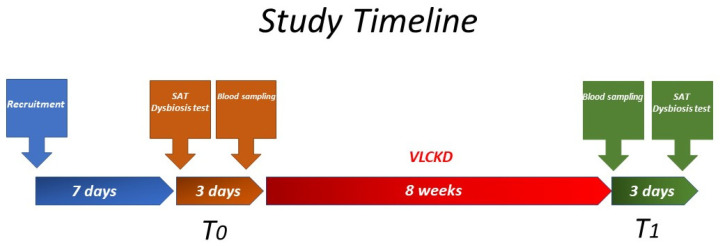
The study design.

Patients with obesity were recruited from April to November 2022. The Gastrointestinal Symptom Rating Scale (GSRS) questionnaire was administered during the recruitment visit as an initial screening [21]. Follow-up visits occurred throughout two medical appointments: before the start of the diet (T_0_) and eight weeks after the beginning of the diet (T_1_) (Figure 1). Before entering the study, all patients underwent s-IP by SAT, which had to produce a Lac to Man ratio below 0.03 as the inclusion criterion. In addition to anthropometric parameters, data relative to fasting blood samples were collected at T_0_ and T_1_. Patients were asked to return after 2–4 days for blood sampling, anthropometric parameters, and the assessment of intestinal dysbiosis. Within 2–4 days after the permeability test, the patients started their personalized VLCKD.

After eight weeks of the diet program, the patients had to repeat blood withdrawal. Lastly, the patients had to undergo the permeability and dysbiosis tests again within three days. 

The study protocol was approved by the internal Medical Ethical Committee (Prot. n. 170/CE De Bellis). The study was performed according to the Helsinki Declaration (1964). Before starting their participation in the study, each patient gave written consent. The ClinicalTrials.gov attributive of the study is NCT05477212. 

### 2.2. Diet Protocol 

The protocol has been previously published in our studies [12] and was strictly in agreement with that described by Bruci et al. [10], but involved only steps 1 and 2 of that protocol. All patients followed a VLCKD plan to replace meals per a 2-step protocol (New Penta, Cuneo, Italy). The total carbohydrate intake was fixed at 20–50 g/day during these two stages. 

Protein and lipids intakes were 1–1.4 g/kg of ideal body weight and 15–30 g per day, respectively. Patients were instructed to consume at least 2 L of water each day. There were fewer than 800 calories consumed each day. In order to avoid nutritional shortages, micronutrient supplements were mandated during the whole dietary therapy [11]. During the first stage, only meal replacements and particular kinds and amounts of vegetables were permitted; however, during the second step, one of the substitute meals was changed to a dish including protein.

The protein dish consisted of fish, white meat, red meat, eggs, and bresaola. As variance, the products from Penta comprised mostly whey proteins as proteins and oleic acid, omega 3 fatty acids, and other polyunsaturated fats as fatty acids; carbohydrates were very little represented, and the type of carbohydrate changed according to the product type. 

The semi quantitative concentration of acetoacetic acid was measured in the first-morning urine at baseline and every week until the end of the study by the patients (Ketur-Test, Accu-Chec, Roche Diagnostics, Monza, Italy).

### 2.3. Anthropometric Parameters and Biochemical Characteristics 

To measure BMI (kg/m^2^), body weight and height were measured in fasting subjects wearing light clothing, barefoot, and with an empty bladder. All patients were measured using the same calibrated scale and stadiometer. Blood samples were taken between 8:00 and 9:00 a.m. after overnight fasting. Standard laboratory procedures were used to measure the biochemical characteristics before and after the VLCKD. 

### 2.4. Sugar Absorption Test 

All participants in the study were evaluated by SAT after an overnight fast. The laboratory’s staff collected a pre-test urine sample to assess the presence of endogenous sugars. After that, patients consumed a solution that contained 10 g of Lac, 5 g of Man, and 40 g of Suc in a volume of 100 mL. Urine was collected for up to 5 h following administration. A 1 mL sample of 20% (*w*/*v*) chlorohexidine was added to every collection as a preservative, regardless of the final volume. The total volume of urine excreted was measured and recorded. After the mixture was thoroughly combined, a sample of 2 mL was removed and stored at −80 °C until analyzed. In urine, the detection and measurement of the three sugar probes, Lac, Man, and Suc, were accomplished via chromatographic analysis, as previously described by our group [22]. The percentages of ingested Lac (%Lac), Man (%Man), and Suc (%Suc) excreted in the urine were evaluated, and the Lac to Man ratio was calculated for each sample as a marker of the small intestinal permeability. Patients with a Lac to Man ratio of 0.030 or higher were categorized as having an altered s-IP [23].

### 2.5. Serum and Fecal Zonulin, Serum I-FABP, and Serum DAO Concentrations

Biochemical assessments were performed on admission and at the end of the diet. Serum samples and crude stool samples from patients in the study were frozen and stored at −80 °C within 12 h of collection. Serum and fecal zonulin were tested by ELISA kit (Immunodiagnostik AG, Bensheim, Germany). According to the manufacturer’s instructions, serum and stool levels below 48 ng/mL and 107 ng/mL were considered normal zonulin levels. I-FABP and DAO serum levels were assessed using ELISA kits (Thermo Fisher Scientific, Waltham, MA, USA) and (Cloud-Clone Corp., Houston, TX, USA). Circulating IL-6, IL-8, IL-10, and TNF-α were measured using ELISA kits (BD Biosciences, Milan, Italy). Lipopolysaccharide (LPS) was measured using the Cloud-Clone Corp. ELISA kit (Katy, TX, USA). 

### 2.6. Indican and Skatole Evaluation

Urine samples were taken in the morning from all patients. According to the manufacturer’s instructions, a typical colorimetric assay kit (Indican Assay Kit, ABNova Corporation, Taipei, Taiwan) was used to measure urine indican levels. Thermo Scientific’s Dionex high-performance liquid chromatography (HPLC) equipment was used to detect and analyze urine skatole using the 3-methylindole kit (EurekaLab Division, Chiaravalle, AN, Italy), as was previously described [18]. Urinary levels of indican and skatole above 20 mg/L and 20 μg/L are considered indicators of fermentative or putrefactive dysbiosis, respectively [24].

### 2.7. Statistical Analysis

No studies of the effect of VLCKD on gut barrier homeostasis have been performed previously, and the present research represents a pilot study. Thus, based on these premises, the statistical power calculation was unnecessary. Statistics were performed using Sigma Stat 11.0 (Systat Software, Inc., San Jose, CA, USA) and GraphPad Prism 8 (GraphPad Software Inc., La Jolla, CA, USA). The Wilcoxon matched-pairs signed rank test was used to detect differences before and after the VLCKD. The Mann–Whitney test was used to assess differences between the two groups. For categorical variables, comparisons were performed using Fisher’s exact test. Unless otherwise specified, all results were expressed as means ± SEM. *p* < 0.05 was considered statistically significant.

## 3. Results

### 3.1. Number, Anthropometric, and Symptomatic Characteristics of the Patients, and Intervention Diet

**Figure 2:**
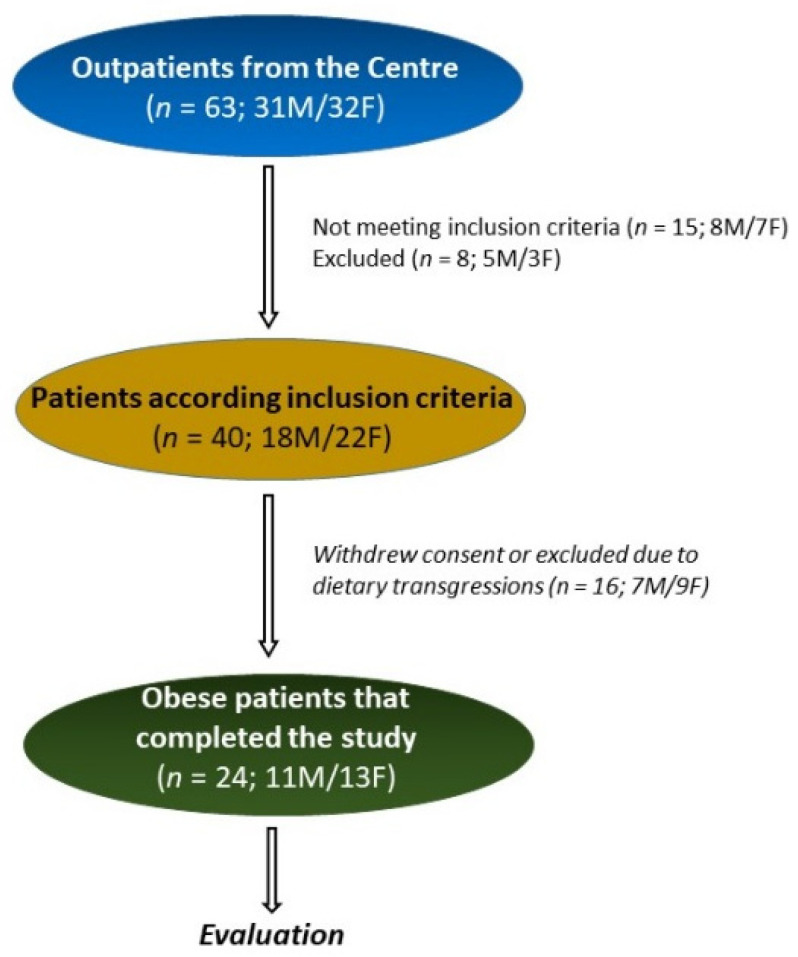
The flow of the patients through the study.

Figure 2 summarizes the flow of the patients throughout the study. In all, 63 patients with obesity were recruited: 31 males (M) and 32 females (F). Of these, 15 patients (8M/7F) did not meet the inclusion criteria, and 8 (5M/3F) patients were excluded for different reasons (pregnancy, abdominal surgery, use of antibiotics, transfer to other districts, change of working activities). Also, 16 patients (7M/9F) withdrew consent or were excluded due to dietary transgressions. Thus, 24 (11M/13F) patients completed this pilot study ny following the diet for 8 weeks. All the patients showed the presence of ketosis in the urine samples collected during the diet. 

Table 1 reports the patients’ anthropometric characteristics before and after VLCKD. Compared to the study’s start, significant decreases in weight, BMI, and waist circumferences were observed at the end of the diet. Both diastolic blood pressure (DBP) and systolic blood pressure (SBP) were significantly lower (*p* < 0.05) after VLCKD. 

Table 2 reports the patients’ biochemical characteristics before and after VLCKD. Fasting blood levels of free triiodothyronine (FT3), free thyroxine (FT4), glucose, insulin, gamma-glutamyl transferase (γGT), alanine aminotransferase (ALT), total cholesterol, high-density lipoprotein (HDL) cholesterol, low-density lipoprotein (LDL) cholesterol, and triglycerides were lower after the VLCKD diet. On the contrary, the 25-OH-Vitamin D blood concentration increased significantly after the VLCKD diet. 

### 3.2. Sugar Absorption Test 

**Figure 3:**
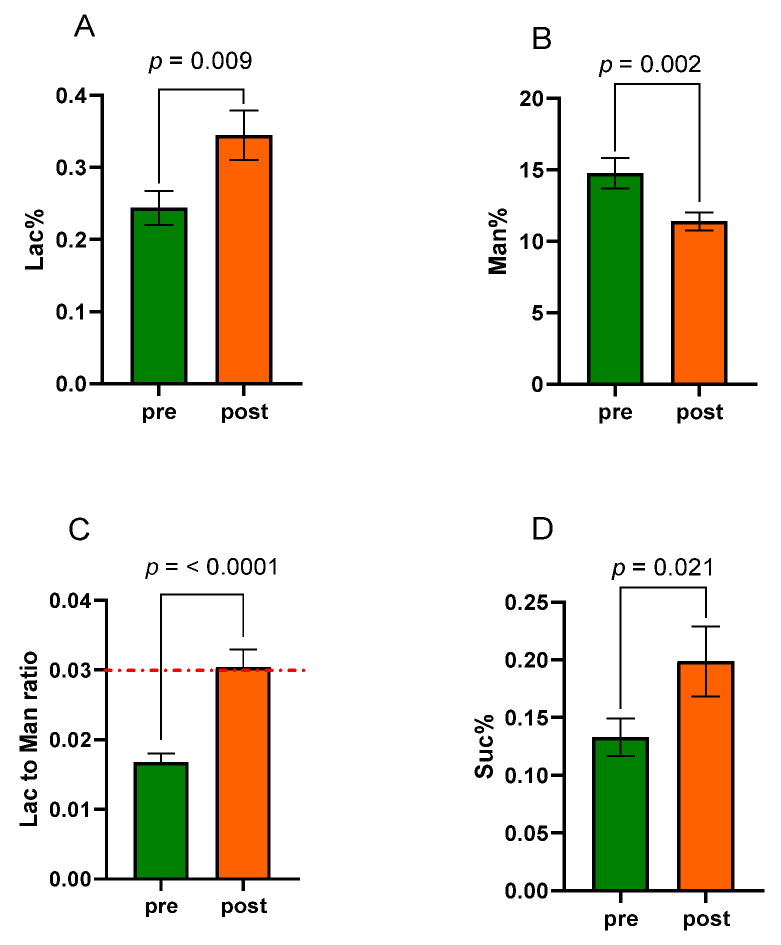
A sugar absorption test (SAT) evaluated the small intestinal permeability (s-IP) before (pre) and after (post) eight weeks of a very-low-calorie ketogenic diet (VLCKD). Lac% = percentage of ingested lactulose excreted in the urine, (**A**). Man% = percentage of ingested mannitol excreted in the urine, (**B**). Lac to Man ratio = lactulose to mannitol ratio, (**C**). Suc% = percentage of ingested sucrose excreted in the urine, (**D**). Data are presented as mean ± SEM. Statistical analysis: Wilcoxon matched-pairs signed-rank test with a significant difference set at *p* < 0.05. The dotted red line indicates the cut-off value for altered small intestinal permeability (Lac to Man ratio ≥ 0.03).

Before entering the study, a sugar absorption test with three sugar probes (lactulose, mannitol, and sucrose) was performed in all patients. The Lac/Man ratio value had to be <0.03 as an inclusion criterion, indicating the absence of altered s-IP. The same test was repeated in all patients after eight weeks of VLCKD. Figure 3 shows the urinary percentages of the three probes Lac% (panel A), Man% (panel B), and Suc% (panel D). The Lac/Man ratio was estimated in all the samples (panel C). 

At the end of the diet, Lac% was significantly (*p* = 0.009) higher than the baseline percentage (0.345 ± 0.034 vs. 0.244 ± 0.023), whereas Man% was significantly (*p* = 0.002) lower than the starting value (11.40 ± 0.62 vs. 14.76 ± 1.07). The Lac/Man ratio increased significantly (0.030 ± 0.002 vs. 0.017 ± 0.001; *p* < 0.0001). Moreover, the Suc% increased significantly at the end of the diet (0.199 ± 0.030 vs. 0.133 ± 0.016; *p* = 0.021). In percent terms, Lac% increased by 41.4% and Man% decreased by 22.8%. The Lac to Man ratio increased by 76.5%. Finally, also the Suc% increased by 49.6% at the end of the diet. 

**Figure 4:**
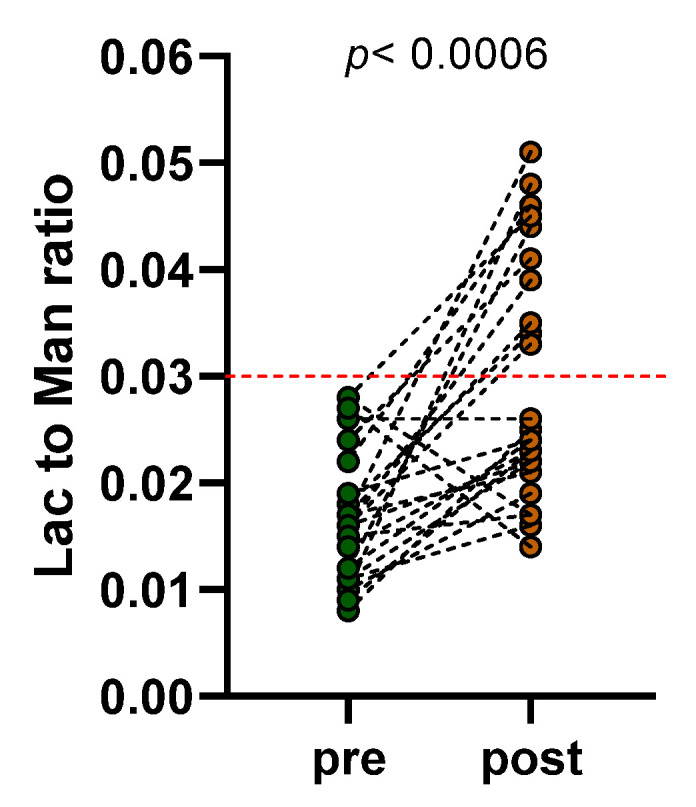
Lactulose to mannitol ratios represented before (pre) and after (post) eight weeks of a very-low-calorie ketogenic diet (VLCKD). Lac to Man ratio = Lactulose to Mannitol ratio. Statistical analysis: Fisher’s exact test with a significant difference set at *p* < 0.05. The dotted red line indicates the cut-off value for altered small intestinal permeability (Lac to Man ratio ≥ 0.03).

Interestingly, not all the subjects in the study showed the same behavior of the Lac to Man ratio following VLCKD. As shown in Figure 4, in 10 (4M/6F) out of 24 subjects with obesity (42%) (Fisher’s exact test, *p* = 0.0006), the ratio increased significantly, with values above the cut-off, indicating an alteration of the s-IP (0.043 ± 0.002 vs. 0.018 ± 0.002, *p* = 0.002). Oppositely, the Lac to Man ratio was not significantly affected by diet in the other 14 patients (7M/7F) and even tended to increase, although not significantly (0.021 ± 0.001 vs. 0.016 ± 0.002, *p* = 0.071). The differences between the two groups relating to the markers of the intestinal barrier, dysbiosis, and bacterial translocation are reported in the following paragraphs.

### 3.3. Biomarkers Related to the Intestinal Barrier Function and Integrity

At the start of the study, mean fecal zonulin concentrations in patients with obesity were above the cut-off level (239.20 ± 23.86 ng/mL). The diet did not modify its concentrations (246.10 ± 30.74 ng/mL; Pre-diet vs. Post-diet, *p* = 0.983), reaching values below the cut-off limit in none of the patients. Similarly, the diet did not affect serum zonulin values (34.67 ± 3.21 ng/mL vs. 39.38 ± 4.01 ng/mL; Pre-diet vs. Post-diet, *p* = 0.129). 

Regarding the integrity of the intestinal barrier, the serum I-FABP concentrations were not different between the start and the end of the study (2.15 ± 0.29 ng/mL vs. 2.31 ± 0.21 ng/mL; Pre-diet vs. Post-diet, *p* = 0.129). Likewise, serum DAO levels were unaffected by treatment (53.96 ± 1.37 ng/mL vs. 55.00 ± 1.91 ng/mL; Pre-diet vs. Post-diet, *p* = 0.284).

The fecal and serum concentrations of the biomarkers of intestinal barrier function and integrity were not different between the start and the end of the study in the subgroups of patients with both impaired and normal s-IP. 

### 3.4. Levels of Inflammatory Factors

**Figure 5:**
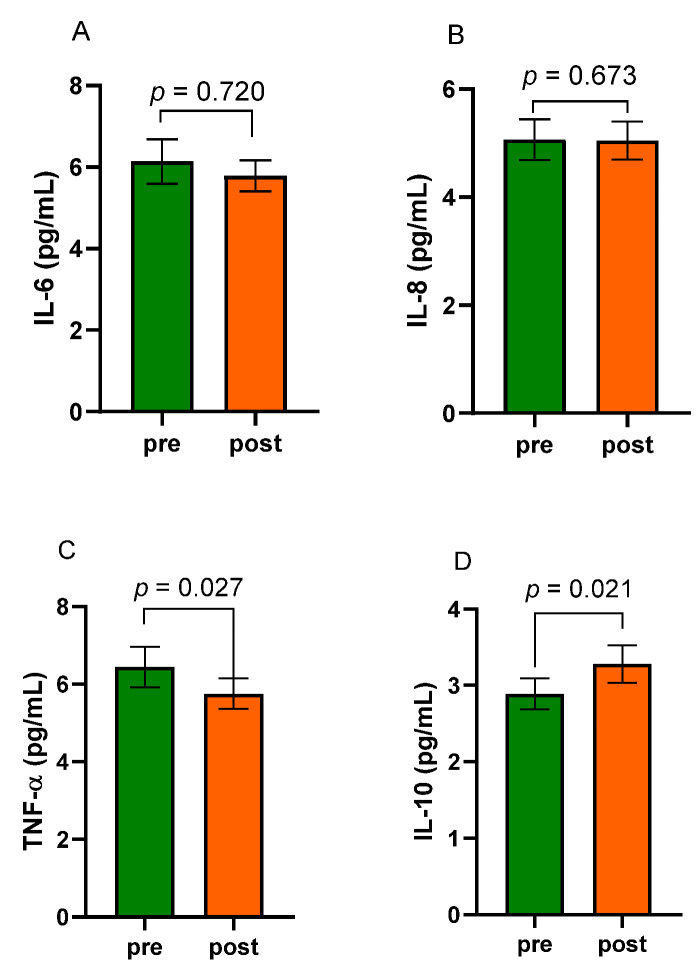
The serum circulating concentrations of interleukin-6 (IL-6, (**A**)), interleukin-8 (IL-8, (**B**)), tumor necrosis factor-α (TNF-α, (**C**)), and interleukin-10 (IL-10, (**D**)) in obese patients before (pre) and after (post) eight weeks of a very-low-calorie ketogenic diet (VLCKD). Data expressed as means ± SEM. Wilcoxon matched-pairs signed-rank test was used to compare pre-treatment and post-treatment data. Differences were considered significant at *p* < 0.05.

Figure 5 reports the circulating concentrations of IL-6 and IL-8 (panels A and B, respectively) and the serum levels of TNF- α (panel C) and IL-10 (panel D) in patients with obesity before and after eight weeks of the VLCKD. 

As for the interleukin circulating levels, both IL-6 and IL-8 were not significantly affected by the diet (IL-6, 6.14 ± 0.54 pg/mL vs. 5.78 ± 0.38 pg/mL; Pre-diet vs. Post-diet, *p* = 0.720. IL-8, 5.06 ± 0.38 pg/mL vs. 5.05 ± 0.35 pg/mL; Pre-diet vs. Post-diet, *p* = 0.673). Notably, TNF-α circulating levels decreased significantly at the end of the treatment (5.75 ± 0.39 pg/mL vs. 6.44 ± 0.52 pg/mL; Post-diet vs. Pre-diet, *p* = 0.027). On the contrary, the anti-inflammatory IL-10 significantly increased its circulating levels at the end of the treatment compared to baseline values (2.89 ± 0.20 pg/mL vs. 3.28 ± 0.24 pg/mL; Pre-diet vs. Post-diet, *p* = 0.021). Also, after the diet, the subgroup of patients with altered Lac to Man ratio showed serum IL-10 levels significantly increased compared to levels before the diet (2.43 ± 0.25 vs. 3.26 ± 0.27 mg/mL; *p* = 0.0098). On the contrary, serum concentrations of the other markers of inflammation did not differ between the start and the end of the study in subgroups of patients with either altered or normal s-IP. Moreover, there were no significant differences in values recorded at the beginning of the VLCKD between the two groups. The same occurred at the end of the diet. 

### 3.5. Intestinal Dysbiosis and Bacterial Translocation

**Figure 6:**
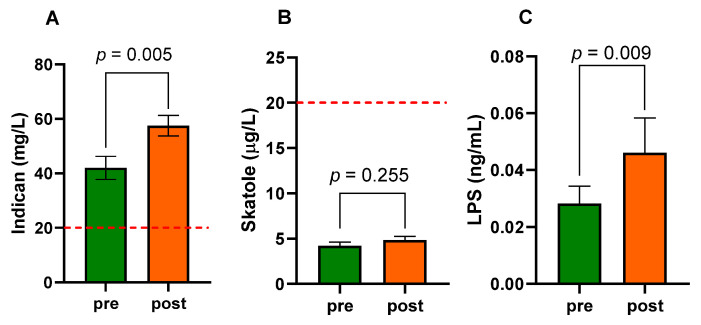
Urinary indican (**A**), urinary skatole (**B**), and serum lipopolysaccharide—LPS (**C**) levels in patients with obesity before (pre) and after (post) eight weeks of VLCKD. Data expressed as means ± SEM. Wilcoxon matched-pairs signed-rank test was used to compare pre-treatment and post-treatment data. Differences were considered significant at *p* < 0.05. The dotted line indicates the cut-off level for dysbiosis: indican (20 mg/L) and skatole (20 μg/L).

At baseline, the urinary indican concentrations in the patients with obesity were approximately twice the level of 20 mg/L (42.00 ± 4.23 mg/L), suggesting the presence of fermentative dysbiosis. At the end of the diet, a significant (*p* = 0.005) increase in urinary concentrations occurred (57.50 ± 3.83 mg/L) (Figure 6A). 

The urinary skatole concentrations were below the level of 20 μg/L, and they were unaffected by diet (4.21 ± 0.42 μg/L vs. 4.83 ± 0.40 μg/L; Pre-diet vs. Post-diet, *p* = 0.255) (Figure 6B). Finally, the concentrations of LPS were significantly higher (*p* = 0.009) at the end of the diet (0.046 ± 0.012 ng/mL) than at the start of the study (0.028 ± 0.006 ng/mL), suggesting an increase in the bacterial translocation (Figure 6C). Interestingly, after the diet, the subgroup of patients with altered Lac/Man ratio had urinary indican and serum LPS levels significantly increased compared to levels before the diet (62.00 ± 6.01 vs. 40.80 ± 8.31 mg/L; *p* = 0.037, and 0.049 ± 0.022 vs. 0.024 ± 0.009 ng/mL; *p* = 0.009, respectively). On the contrary, the urinary indican and serum LPS levels were not different between the start and the end of the study in the subgroup of patients with normal s-IP. 

## 4. Discussion

Obesity is a major health problem affecting millions of persons worldwide, and diet often represents a therapeutic approach for managing these patients. VLCKD is now widely used for weight loss and is regarded as an efficient and secure therapeutic intervention for people affected by obesity [9]. Present results, corroborated by our previous research [12], demonstrate that VLCKD significantly improves metabolic biomarkers and anthropometric and body composition parameters. 

Interestingly, obesity has often been associated with impaired intestinal barrier function, which may facilitate the entry of dietary or microbial antigens, resulting in chronic inflammation, tissue damage, and allergies [25,26]. Concerning the KD effects on intestinal barrier homeostasis, recent data in animal models have reported conflicting results [27,28], but there are no data about humans. Moreover, to our knowledge, no studies on the evaluation of IP alterations in subjects with obesity undergoing VLCKD are available in the literature. 

In this pilot study, we evaluated s-IP, the urinary; serum, and fecal markers of GI barrier function and integrity; and dysbiosis in subjects with obesity and analyzed the effect of eight weeks of VLCKD on these parameters. IP evaluation, particularly the s-IP, is a diagnostic measure of intestinal barrier function [14]. In animal models of obesity, increased IP was associated with elevated endotoxemia and alterations in glucose metabolism [29], while results from studies in obese subjects are still inconsistent [30]. 

At the start of the study, although all patients showed normal s-IP, as evaluated by Lac to Man ratio, the mean fecal zonulin and urinary indican levels, which indicate fermentative dysbiosis, were twice as high as the cut-off values. Our results agree with recent research performed in a group of professional athletes that showed no statistically significant association between fecal zonulin and increased IP [31]. Additionally, Brignardello et al. [32] reported previously that asymptomatic individuals with obesity did not have evidence of gut barrier alterations, despite the influence on the biodiversity of their intestinal microbiota. Another study comparing two groups of women with and without obesity observed that although lactulose and mannitol urinary excretions were higher in women with obesity, the Lac to Man ratio did not differ statistically significantly across the study groups. However, higher insulin and low-density lipoprotein/high-density lipoprotein (HDL) concentrations and lower HDL concentrations were linked to higher lactulose/mannitol ratios. These findings imply that obesity and metabolic syndrome may be linked to intestinal barrier function [3].

Moreover, it has been suggested that intestinal permeability changes in obesity may be a late event in older individuals [14,33]. All these data allow us to hypothesize that alterations of zonulin, microbiota, and intestinal permeability may not necessarily be events always strictly linked by cause and effect. In patients with obesity, this link could also be affected by differences in the characteristics of the subjects, such as weight and age, or their dietary habits, particularly fat content. About this last point, among the different available nutritional strategies, VLCKD, a diet with a very high percentage of fat, has been proposed as an appealing option for obesity management [8].

An important finding of our study shows that, after eight weeks of VLCKD, considering the patients as a whole group, the Lac to Man ratio increased significantly compared to baseline values. However, this behavior was not common for all the patients since, at the end of the diet, 10 out of 24 had an increased Lac to Man ratio above the threshold value for altered intestinal permeability, while 14 out of 24 patients still showed normal values. Interestingly, the other intestinal barrier function and integrity markers were unaffected by VLCKD, since they did not significantly change after the diet in the whole group or subgroups divided by altered intestinal permeability. Nonetheless, in the whole group, the VLCKD diet decreased TNF-α and increased IL-10 levels, confirming its anti-inflammatory properties, as reported by other researchers [34,35,36].

Additionally, the whole group and the subgroup of patients with impaired s-IP had urinary indican and serum LPS levels significantly higher after the diet than baseline. On the contrary, the urinary indican and serum LPS levels did not change significantly after diet in the subgroup of patients with normal s-IP. The last two parameters indicate a modification of the balance conditions between the intestinal lumen and microbiota.

The dietary components may significantly influence the gut microbiota, affecting its richness and diversity in terms of composition, according to some research. On the one hand, a high diet of animal proteins, saturated fat, sugar, and salt could promote the proliferation of harmful bacteria at the expense of helpful bacteria, perhaps causing changes to the intestinal barrier. On the other hand, ingesting complex polysaccharides and plant protein may be linked to an increase in the number of beneficial bacteria, which in turn stimulates the creation of SCFA, which is essential for maintaining the integrity of the intestinal barrier [37].

Rondanelli et al. [38] described the effects of VLCKD on the gut microbiota, reporting that a reduction in carbohydrate intake, as is characteristic of VLCKD, can lead to a decrease in polysaccharide content in the gut, which in turn can lead to a decline in bacterial benefits of the intestinal microbiota such as bifidobacteria. Conversely, other bacterial strains, such as *Akkermansia* or *E. coli*, can increase during a ketogenic diet, adversely affecting gut health.

Although it did not fall within the aims of the present research, the results of this study allow us to hypothesize that the increased levels of indican and LPS reflect the effects of diet on microbiota and s-IP. However, it is conceivable that this effect may not occur to the same extent in all obese patients. Future studies will be performed by profiling the intestinal microbiota of these patients to shed light on this issue. Accumulating evidence suggests that dysregulated microbiota and their metabolites caused by a diet with a higher percentage of fat can lead to intestinal mucosal barrier dysfunction by affecting the programmed death of intestinal epithelial cells, reducing the secretion of goblet cells and Paneth cells, impairing intercellular connections, and disrupting the immune balance [39].

While the VLCKD diet can show a positive impact on the weight of patients with obesity, as well as represent a helpful approach for NAFLD [12], its collateral effects on the gut microbiota composition and function, as well as integrity of the gut barrier, may raise concerns. VLCKD may likely represent a triggering factor capable of worsening a compromised intestinal balance, such as that in patients with obesity.

Thus, for future research, it will also be mandatory to assess whether these changes in intestinal barrier function and integrity persist over time, as they may only be temporary. If so, they should be considered a small price to pay for the immediate beneficial effects of the ketogenic diet on the weight and metabolism of patients with obesity.

This study has some weaknesses. Firstly, the present results are derived from a pilot study, so the size of the tested population prevents us from drawing firm conclusions. Secondly, the onset of GI symptoms, impaired s-IP, and the role of the indices of mucosal integrity need further study. Third, the suggested fermentative dysbiosis found with urinary indican evaluation was not supported by data from other, more appropriate methods because bacterial populations in the GI tract have not been adequately analyzed (i.e., by molecular analysis of the 16S rRNA gene). Another weakness of this study is that we did not measure the ketogenic ratio of the diet, as suggested by Zilberter et al. [40]. However, our model of the ketogenic diet has been previously published in several studies [10,12] and respects the European Guidelines for Obesity Management in Adults with a Very Low-Calorie Ketogenic Diet [8]. Lastly, urine samples during the diet showed the presence of ketosis in all the patients. Measuring capillary blood concentrations of b-hydroxybutyrate would have been a more accurate method of ketosis assessment than the urinary acetoacetate semi-quantitative determination used in the study for technical reasons. However, the fundamental objectives that our study had set were achieved. Further research is needed to investigate the still-unveiled aspects linking obesity, intestinal barrier alterations, and VLCKD.

## 5. Conclusions

Overall, the evidence provided here suggests a relationship between the intestinal barrier, the ketogenic diet, and its impact on the gut microbiota in patients with obesity. The main finding of this study shows that obese patients, after 8 weeks of the VLCKD diet, presented a compromised intestinal barrier, dysbiosis, and elevated serum levels of LPS. However, this behavior was not common for all the patients. Therefore, while the VLCKD diet can show a positive impact on the weight of patients with obesity, its effects on the gut microbiota composition and function, as well as the integrity of the gut barrier, may raise concerns. VLCKD may likely represent a triggering factor capable of worsening a compromised intestinal balance, such as that in patients with obesity. Further research is needed by recruiting more patients and extending the observation time to fully understand the role of the intestinal barrier and gut microbiota variations during VLCKD.

## Figures and Tables

**Table 1:** Anthropometric characteristics of the patients with obesity before (Pre) and after (Post) 8 weeks of a very-low-calorie ketogenic diet (VLCKD).

	Pre-VLCKD (*n* = 24)	Post-VLCKD (*n* = 24)	*p*
Age (years)	42.58 ± 2.64	42.58 ± 2.64	ns
Sex (M/F)	11 M/13 F	11 M/13 F	ns
Weight (Kg)	101.80 ± 5.18	92.76 ± 4.95	<0.0001
Height (m)	150.60 ± 11.85	150.60 ± 11.85	ns
BMI (Kg/m^2^)	35.56 ± 1.12	31.56 ± 1.30	<0.0001
Waist circumference (cm)	111.80 ± 3.48	102.80 ± 3.63	<0.0001
DBP (mmHg)	86.33 ± 2.47	79.29 ± 1.54	<0.0001
SBP (mmHg)	137.40 ± 3.19	127.00 ± 1.08	0.0002

BMI: Body Mass Index; DBP: diastolic blood pressure; SBP: systolic blood pressure. Data are expressed as means ± SEM and analyzed by Wilcoxon matched-pairs signed-rank test. All differences were considered significant at *p* < 0.05. ns: not significant.

**Table 2:** Biochemical characteristics of the patients with obesity before (Pre) and after (Post) 8 weeks of a very-low-calorie ketogenic diet (VLCKD).

	Pre-VLCKD (*n* = 24)	Post-VLCKD (*n* = 24)	*p*
TSH (μU/mL)	1.85 ± 0.26	1.90 ± 0.17	ns
FT_3_ (pg/mL)	3.38 ± 0.06	2.98 ± 0.09	<0.0001
FT_4_ (pg/mL)	10.73 ± 0.41	11.90 ± 0.43	0.0002
Glucose (mg/dL)	98.63 ± 3.83	91.08 ± 2.85	0.0027
Insulin (μU/mL)	16.08 ± 2.15	10.54 ± 1.10	<0.0001
Uric acid (mg/dL)	5.55 ± 0.28	5.79 ± 0.27	ns
25-OH-Vitamin D (ng/mL)	21.67 ± 1.76	25.71 ± 2.37	0.0002
γGT (U/L)	27.63 ± 4.20	19.08 ± 2.70	<0.0001
ALT (U/L)	36.79 ± 5.75	31.79 ± 6.70	0.033
AST (U/L)	24.67 ± 2.46	22.50 ± 2.48	ns
Total Cholesterol (mg/dL)	207.10 ± 8.41	180.20 ± 5.85	<0.0001
HDL Cholesterol (mg/dL)	55.78 ± 3.52	50.74 ± 2.97	0.0060
LDL Cholesterol (mg/dL)	141.40 ± 7.62	113.00 ± 5.93	<0.0001
Triglycerides (mg/dL)	109.80 ± 14.06	93.46 ± 9.81	0.0010

TSH: thyroid-stimulating hormone; FT_3_: free triiodothyronine; FT_4_: free thyroxine; γGT: gamma-glutamyl transferase; ALT: alanine aminotransferase; AST: aspartate aminotransferase. Data are expressed as means ± SEM and analyzed by Wilcoxon matched-pairs signed-rank test. All differences were considered significant at *p* < 0.05. ns: not significant.

## Data Availability

The datasets used and/or analyzed during the current study are available from the corresponding author upon reasonable request.
